# Cross-Sectional Observational Study of the Differences in Cephalometric Parameters in German Class I/II Orthodontic Patients

**DOI:** 10.1155/ijod/9665260

**Published:** 2025-08-21

**Authors:** Eva Paddenberg-Schubert, Kareem Midlej, Sebastian Krohn, Iqbal M. Lone, Osayd Zohud, Obaida Awadi, Samir Masarwa, Aysar Nashef, Christian Kirschneck, Nezar Watted, Peter Proff, Fuad A. Iraqi

**Affiliations:** ^1^Department of Orthodontics, University Hospital of Regensburg, University of Regensburg, Regensburg 93047, Germany; ^2^Department of Clinical Microbiology and Immunology, Faculty of Medicine and Health Sciences, Tel Aviv University, Tel Aviv 6997801, Israel; ^3^Center for Dentistry Research and Aesthetics, Jatt 4491800, Israel; ^4^Department of Oral and Maxillofacial Surgery, Meir Medical Center, Affiliated With the Faculty of Medici Medicine and Health Sciences, Tel-Aviv University, Kfar Saba, Israel; ^5^Department of Orthodontics, University of Bonn, Bonn D-53111, Germany; ^6^Gathering for Prosperity Initiative, Jatt 4491800, Israel; ^7^Department of Orthodontics, Faculty of Dentistry, Arab America University, Jenin, State of Palestine

**Keywords:** cephalometric analysis, correlation, personalised orthodontics, principal component analysis, skeletal malocclusion

## Abstract

**Objectives:**

The correct classification of orthodontic patients is essential in individualized diagnostics and treatment planning. However, due to the complexity of the craniofacial skeleton and differences related to gender, age, and ethnicity, cephalometric analysis can be prone to errors. This multicenter, cross-sectional study aimed to compare cephalometric measurements between skeletal class I and II in German orthodontic patients and analyze the effect of gender/age subgroups.

**Materials and Methods:**

In total, 556 German orthodontic patients were included and stratified into skeletal class I (*n* = 210) and II (*n* = 346), based on the individualized ANB of Panagiotidis and Witt (Calculated_ANB). Both classes presented a mean age of 13 with a range of 6.6–41 years and 5.4–53 years in classes I and II, respectively. Regarding the gender variations, most participants were females, *n* = 194 (56%) among class I, and *n* = 125 (60%) among class II. Cephalometric parameters were compared between classes and among age and gender-specific subgroups, followed by identifying correlations and performing principal component analysis (PCA).

**Results:**

Class II patients presented a more considerable sagittal discrepancy between jaw bases than class I cases (Calculated_ANB 2.8° vs. 0.025°), a more horizontal growth pattern (Gonion angle 119° vs. 123°), and compensated inclinations of the incisors in the upper (+ 1/NL 71° vs. 68°) and lower jaw (−1/ML 84° vs. 80°). Correlations were found between sagittal, vertical, and dental cephalometric parameters, which were strongest in adult class II males. Finally, ML-NSL angle, SNPg angle, PFH/AFH ratio, and SNB angle are related to the variations of the first four components.

**Conclusions:**

The differences in cephalometric parameters between skeletal class I and II demonstrate certain configurations in vertical, sagittal, and dental parameters, and identifying these marks precisely will enable accurate diagnosis. In addition, the variations concerning gender and age highlight the possible influence of these factors on orthodontic diagnostics and treatment planning. Future studies with equal sample sizes among subgroups must validate these findings. Finally, the PCA results highlighted that the mandible's vertical and sagittal position has a strong influence on the diagnosis of skeletal class I/II, which highlights the importance of identifying the corresponding reference marks.

## 1. Introduction

One of the critical responsibilities of orthodontics is the identification of physiological and pathological jaw positions, that is, the distinction between eugnathic and dysgnathic relations of the maxilla and mandible, and the treatment of such pathologic craniofacial structures [1]. Hence, as in all medical disciplines, it is of major importance to the orthodontic practitioner to perform precise and correct diagnostics and to evaluate the orthodontic treatment needs for each patient individually [1]. The diagnostic procedures and treatment planning can be categorized by the kind of craniofacial structures affected, that is, anomalies of the jaws, teeth, dentition, and stomatognathic functions [1]. In the case of dysgnathia, which refers to an abnormal position, relation, and/or size of the jaw bases, treatment options vary concerning the patient's age [2]. Growing patients can benefit from functional orthopedic appliances [3], while treatment options for adults include dental compensation in terms of camouflage therapies or combined maxillofacial surgical-orthodontic treatment strategies [4].

Routine orthodontic diagnostics are comprised of anamnesis, photographs from intra- and extraoral, clinical orthodontic examination, dental cast analysis, and radiographic examination, which often consists of an orthopantomogram and a lateral cephalogram [5]. Among others, cephalometric analysis determines the skeletal class, defined as the relation of the maxilla and mandible in the sagittal direction [6]. It can be categorized into two classes: in skeletal class I, the upper and lower jaw are neutrally oriented to each other [7], whereas in class II, the mandible presents a pathologically posterior position in relation to the maxilla [8]. The prevalence of skeletal class II is relatively high, although variations are found among different populations. For example, up to 29% of the worldwide population present class II and up to 62% class I, although differences are observed between various ages and ethnicities [9]. Hence, the correct diagnosis of skeletal class II patients compared to an “ideal” desirable skeletal class I is of crucial interest to the orthodontic practitioner.

There are various methods to define a patient's skeletal class, including “classical” approaches such as the ANB angle described by Riedel [10] and individualized techniques like the graphical procedure suggested by Fishman [11], the harmony box established by Segner and Hasund [12, 13], or the individualized ANB introduced by Panagiotidis and Witt [14]. In classical approaches, empirical norms are used as a reference during interpretation of the measurements, which bears the risk of false diagnoses since the intraindividual harmony of the craniofacial skeleton is not considered. Such relations of anatomical structures, which develop during growth, were already described by Enlow et al. [15]. Several studies have already identified correlations among specific parameters. In a study that was done by Pillai et al. [16], and examined the association of the gonial angle with age, gender, and dental status, using lateral cephalogram and orthopantomogram, found that a significant negative correlation was observed between gonial angle and age in the orthopantomograms. Besides, the gender difference was significant in cephalograms. In another study that was consisted of 250 elderly subjects of both genders, and examined cephalometric characteristics in the elderly, found that the cephalometric measurements females were significantly lower. In addition, a significant decrease in the cephalometric values was observed in relation to the growth pattern, with the advancement of age, and finally, ethnic groups (white, black, and Japanese) showed significant variations in the cephalometric parameters [17]. These are considered in individualized approaches, which define a floating norm value for each patient, based on guiding variables. Many authors have described this method, resulting in various available techniques. A very common method for such floating norms in skeletal class diagnosis is the individualized ANB angle of Panagiotidis and Witt [14], which is a regression equation. It includes the inclination of the mandibular plane and the degree of prognathism of the maxilla, thereby resulting in higher precision of an individual's true skeletal class. Although individualized methods in cephalometry present advantages in terms of precision, differences with respect to age, gender, and population must be considered [18–21]. According to our knowledge, differences with respect to age and gender were not comprehensively studied among skeletal class I and II German population.

Recently, we published a paper that consisted of skeletal class I and II German population, and was dedicated to improve the skeletal classification using machine-learning models based on minimal cephalometric parameters [22]. However, this paper focuses on two primary aims. First, we applied individualized ANB norms (floating norms), and second aim was to assess their relevance in distinguishing skeletal class I versus II in a German population, which has not been sufficiently explored in previous literature, mainly to examine gender and age-specific subgroups effects and to analyze the correlation between those variables.

## 2. Materials and Methods

### 2.1. Ethical Statement

Before sample collection, this investigation received ethical approval from the University of Regensburg (Approval Number 19-1596-101, 13/11/2019). The study recruited patients exclusively from German specialist orthodontic offices and the Department of Orthodontics at the University Hospital Regensburg, Germany. The study adhered to the declarations of Helsinki and the ethical guidelines approved by the university's committee.

### 2.2. Sample Size

The sample size was determined by the maximum available German patients that were diagnosed as skeletal class I or II within the recruitment period. Besides, we calculated the estimated sample size needed needed if we suggest a moderate correlation (*p* = 0.5) in the different subgroups of gender and age, using the formula [23]:

  $$\begin{array}{c} \text{Total sample size }=N=\;\left[\left(Z\alpha +Z\beta \right)/C\right]2+3, \end{array}$$

  $$\begin{array}{c} \text{The standard normal deviate for }\alpha =Z\alpha =\;1.9600, \end{array}$$

  $$\begin{array}{c} \text{The standard normal deviate for }\beta =Z\beta =\;0.8416, \end{array}$$

  $$\begin{array}{c} C=\;0.5\times \ln\left[\left(1+r\right)/\left(1-r\right)\right]=0.5493. \end{array}$$

The estimated sample size needed for moderate correlation was 29 patients (for each subgroup).

### 2.3. Cephalometric Analysis

This study was based on pretreatment lateral cephalograms of German orthodontic patients taken as part of their routine diagnostics. The clarification criteria were the availability of a pretreatment lateral cephalogram with caliper for calibration, demographic information (age and gender), and the presence of either skeletal class I or II as diagnosed by the individualized ANB.

Patients were stratified into the groups skeletal class I and II and, secondly, into age (≤13 years, 14–21 years, and >21 years) and gender-specific subgroups:

Lateral cephalograms were digitized if necessary and imported as lossless TIF files into the software ivoris analyze pro (Computer konkret AG, Falkenstein, Germany, version 8.2.15.110) for calibration and cephalometric analysis. The individualized ANB was calculated using the formula suggested by Panagiotidis and Witt:

• Individualized ANB [14] = −35.16 + (0.4 × SNA) + (0.2 × ML-NSL).
• Calculated_ANB = ANB_measured− ANB_individualised.

To avoid data distortion from borderline cases, the limits were set slightly broader than the original range of ±1°:

• Skeletal class I: −1.5°≤Calculated_ANB≤1.5°.
• Skeletal class II: Calculated_ANB >1.5°.
• Skeletal class III: Calculated_ANB <−1.5°.

A comprehensive cephalometric analysis, similar to Segner and Hasund, was performed, evaluating skeletal sagittal, skeletal vertical, and dental parameters listed in Table S1 and illustrated in Figure S1.

### 2.4. Data Analysis

Interrater- and intrarater reliability were verified using the test–retest method. All other statistical analyses were performed with the R software (https://www.r-project.org/). In detail, differences in cephalometric measurements between groups were analyzed by Kruskal–Wallis with post hoc Dunn analysis and Bonferroni correction. Besides, correlation among parameters was assessed by Spearman correlation as we conducted in our earlier study [24]. Principal component analysis (PCA) was evaluated using the scree plot, component explained in each component, and the cosine square function and loading plot. Prior to performing the PCA analysis, we performed Kaiser–Meyer–Olkin (KMO) test, in order to check the data adequacy for factor analysis. The initial results showed overall measurement systems analysis (MSA) = 0.5, which is not fully accepted for performing factor analysis. Therefore, we removed the ANBind, which is redundant with the Calculated_ANB (ANB− ANBind), and the updated MSA was 0.74.

Statistical significance and high significance were set at *p* < 0.05.

## 3. Results

### 3.1. Patients

The final study collective comprised 556 German orthodontic patients, stratified to skeletal class II (*n* = 210) and I (*n* = 346). Both groups presented a mean age of 13 with a range of 6.6–41 years and 5.4–53 years in classes I and II, respectively. Further details concerning the demographic information are shown in Table S2. The descriptive statistics of the cephalometric parameters are presented in Table 1.

### 3.2. Multiple Comparisons

Detailed results of the cephalometric comparisons performed are presented in the Tables S3–S7, whereas in the Tables 2–4, only the comparisons with the biggest significant differences are shown, especially substantial differences between groups of the same phenotype (e.g., male vs. male).

The most relevant, significant differences in cephalometric parameters between various subgroups within each skeletal class group are presented in Table 2. In the vertical direction, adolescents had a more horizontal growth pattern (PFH/AFH 2.2% bigger and Gonion angle 2.8° smaller) and hypodivergent jaw bases (NL/ML 2.3° smaller) than children. Among the dental parameters, only the inclination of the upper incisors (+1/NSL and +1/NA) was significantly different between groups: a higher proclination was detected in males (+1/NSL 2° smaller and +1/NA 1.8° bigger) and children (+1/NA 3.5° smaller). Regarding skeletal class II, no vertical parameters proved to be significantly different between skeletal class II subgroups. In adults, especially in males, upper incisors were more retroinclined (+1/NL, +1/NSL, and +1/NA) than in growing patients.

In Table 3, the most relevant significant differences in skeletal cephalometric variables between various age and gender specific subgroups of skeletal class II and I patients are reported, while in Table 4, we summarize the differences in dental cephalometric parameters among the different subgroups of skeletal class I and II.

### 3.3. Correlation Analysis

**Figure 1:**
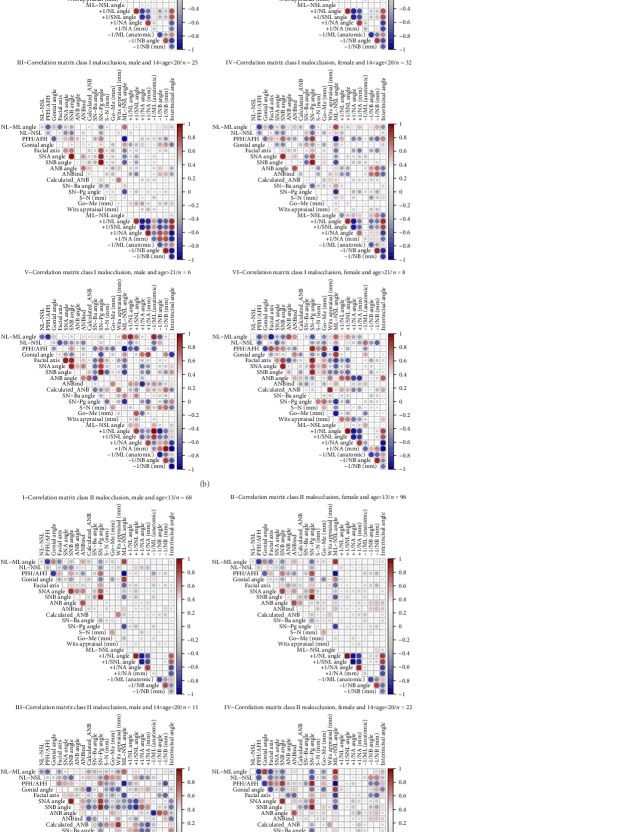
(A) Heatmap correlation matrices demonstrating the correlation between sagittal, vertical, and dental parameters for skeletal class I and II patients. (B) Heatmap correlation matrices demonstrating correlations between cephalometric parameters for different age and gender specific subgroups of patients presenting skeletal class I. (C) Heatmap correlation matrices of cephalometric parameters for different age and gender specific subgroups of patients presenting skeletal class II.

The correlation between cephalometric parameters was evaluated using Spearman correlation tests and their graphical illustration as heatmap matrices. In Figure 1A, the correlation matrices for the total skeletal class I and II are shown. Regarding skeletal class I patients (Figure 1A-I) in the vertical direction, NL-ML was negatively correlated with PFH/AFH (*ρ* = −0.733 and *p*  < 0.01), and positively with ML-NSL (*ρ* = 0.800 and *p*  < 0.01), showing that the inclination of the lower jaw (ML-NSL) was positively associated with the divergence of the jaw bases (NL-ML), which in turn was negatively related to the growth pattern (PFH/AFH). Furthermore, the growth pattern (PFH/AFH) was negatively correlated with the inclination of the mandible (ML-NSL) (*ρ* = −0.955, *p*  < 0.01). Linking the vertical and sagittal direction, ML-NSL was negatively associated with the degree of prognathism of the mandible (SNB) (*ρ* = −0.740, *p*  < 0.01), and with the sagittal position of the chin (SN-Pg) (*ρ* = −0.813, *p*  < 0.01). Concerning the sagittal parameters only, SNA presented a positive correlation with SNB (*ρ* = 0.893, *p*  < 0.01) and SN-Pg (*ρ* = −0.808, *p*  < 0.01), showing the association between the sagittal position of both jaw bases and the chin. Moreover, SNB was positively correlated with SN-Pg (*ρ* = 0.964, *p*  < 0.01), which demonstrates the sagittal relation between the lower jaw and the chin. Overall, similar observations were made for skeletal class II patients (Figure 1A-II).

In Figure 1B (I–VI) the correlations matrices of cephalometric parameters are presented for different age and gender specific subgroups of patients with skeletal class I. First, it becomes obvious that the amount and degree of correlations rose at higher ages for both male and female patients, although the biggest sample size was achieved in children (age <13). For example, among females, Calculated_ANB was correlated moderately with Wits appraisal in children (*ρ* = 0.456, *p*  < 0.01) and adolescents (*ρ* = 0.462, *p*  < 0.01), but in adults: here, a positive correlation with Wits appraisal was found (*ρ* = 0.881, *p*  < 0.01). Many dental parameters revealed correlations with each other (*p*  < 0.05), although they were affected by gender and age: the most and strongest associations were identified in male adolescents, whereas female and young patients presented fewer. Dental parameters, which are intended to measure the same, were correlated with each other, such as −1/ML and −1/NB (*ρ* = −0.762, *p*  < 0.05) or +1/NL and +1/NSL (*ρ* = 0.952, *p*  < 0.01) in female adults. Furthermore, it can be summarized that the upper and lower incisors tended to be inclined into the same direction.

Figure 1C (I–VI) shows the correlation matrices of cephalometric variables for different subgroups of skeletal class II patients. As in class I, gender and age influence the associations since the amount and degree of correlation increased as age rose and because males presented slightly more correlations than females. However, in the class II group, the number of adult subgroups was also the smallest. Regarding Calculated_ANB, the most significant and relevant correlations were identified in male adults, in whom the strongest were Wits appraisal (*ρ* = 1.00, *p*  < 0.01), PFH/AFH (*ρ* = 0.829, *p*  < 0.05), facial axis (*ρ* = 0.886, *p*  < 0.05) (positive) and ML-NSL (*ρ* = −0.829, *p*  < 0.05), NL-ML (*ρ* = −0.829, *p*  < 0.05), Gonion angle (*ρ* = −0.943, *p*  < 0.05) (negative). Whereas the association with Wits appraisal demonstrates the similarity of different parameters, which both aim to determine skeletal class, the others clarify the dependance between sagittal and vertical parameters. Compared to the heatmaps of skeletal class I subjects (Figure 1B), class II patients showed less and weaker correlations between dental variables: here, strong associations were identified for parameters measured in the same jaw or interincisal angle, but not for variables, which include both jaws.

### 3.4. PCA

**Figure 2:**
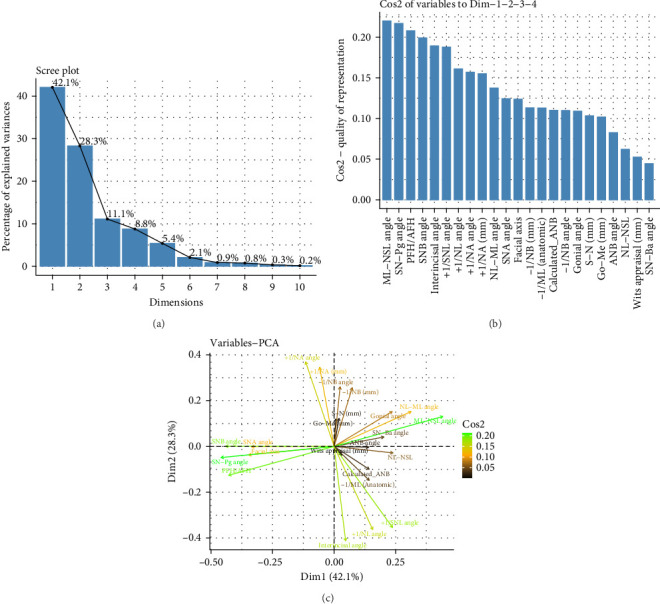
(A) Scree plot result of the principal component analysis. The *x*-axis shows the components, while the *y*-axis represents the percentage of explained variance. (B) Cosine squared function demonstrating the proportion each cephalometric parameter contributes to the first four components of principal component analysis. (C) Loading plot demonstrating the contribution of cephalometric parameters assessed on the first four components of principal component analysis.

PCA was conducted to identify the four most important principal components that are necessary to correctly classify an individual patient as skeletal class I or II. The findings are presented in Table 5 and in the scree plot in Figure 2A, and they reveal, that 90% of the total variance could be explained by the first four components, whereas almost half of the variance (40%) could be attributed to the first component.

Further investigations aimed to identify the loadings of those four principal components and the corresponding results are shown in Table 6. The first principal component was mainly influenced by four sagittal and vertical skeletal parameters, that is SNB (−0.37), SN-Pg (−0.38), PFH/AFH (−0.36) and ML-NSL (0.37). In contrast, the second principal component was mainly affected by dental variables (+1/NL −0.37, +1/NSL −0.36, +1/NA 0.38, +1/NA [mm] 0.35, interincisal angle −0.42). Cephalometric parameters with a high loading on the third principal component were ANB angle (−0.31), Calculated_ANB (−0.43), Wits appraisal (−0.36), and −1/ML (0.36). Finally, the fourth component was mainly influenced by S-N (mm) (−0.48), Go-Me (mm) (−0.48), +1/NA (mm) (−0.32), and −1/NB angle (0.33).

The cosine squared function, which is illustrated in Figure 2B, and the loading plot, which is presented in Figure 2C, show the proportion, which each of cephalometric variable assessed contributed to the first four principal components of the PCA, conducted to correctly classify a patients as skeletal class I or II. According to these figures, the most relevant cephalometric parameter was the inclination of the mandible (ML-NSL) with a cos2 of more than 0.20, followed by the variables SN-Pg, PFH/AFH and SNB, which were directed into the opposite direction (Figure 2C).

## 4. Discussion

This study aims to investigate differences in cephalometric variables concerning age, gender, and skeletal malocclusion with multiple comparison tests and to explore the correlations between the different cephalometric parameters within specific German gender and age groups. For this purpose, as a secondary outcome, PCA was performed to identify the most relevant parameters contributing to skeletal class I and II variance, as was done in our previous study on analyzing the cephalometric parameters of class II and III in the Arab ethnic group [24].

### 4.1. Cephalometric Measurements

Diagnostic cephalometric parameters (Calculated_ANB, ANB, Wits appraisal), were higher in skeletal class II patients compared to skeletal class I, which confirms the correct allocation of patients to the two study groups [14]. SNA was not significantly different between class I and II, which was also found by Jacob and Buschang [25]. However, SNB and SNPg were smaller in class II subjects, indicating that the sagittal position of the mandible appeared to have a bigger effect on skeletal class than the upper jaw. This effect was reinforced in male adults, illustrating that the degree of a dysgnathia can worsen during growth [26]. Furthermore, especially in class I, high ranges between the minimum and maximum were found for SNA and SNB, which clarifies that various combinations can result in an ideal sagittal relation of the jaw and, hence, the need for floating norms to determine an individual's skeletal class [12, 13]. In the vertical direction, class II cases had a more horizontal growth pattern according to Gonion angle, especially in female adults, although facial axis indicated a slightly more vertical growth pattern in class II. Considering the size of the differences, they were clearly higher in Gonion angle (−3.5° in females), so that the contradicting finding of facial axis might be neglected due to the comparatively small size (−1.9° in females). Moreover, among males and children, the upper jaw was more posterior rotated in class II cases compared to class I patients, but the small size of the difference (1.7° in males) questions the clinical relevance. In contrast to our results, Stahl et al. [26] and Baccetti et al. [27] did not report significant differences in vertical skeletal parameters between class I and class II division I patients. Possible explanations for this contrast might be the differences in the population and in the cephalometric parameters assessed. Dental parameters indicated a dental compensation of the skeletal dysgnathia, because patients with class II presented more retroinclined and retropositioned upper and proclined lower incisors than class I subjects. As in sagittal skeletal parameters, these differences were biggest in adults in the upper jaw, but in children and adolescents in the lower jaw. Hence, this finding might indicate that dental compensation of severe skeletal class II in adults is mainly driven by the upper incisors. This observation is confirmed by Steiner, who explained that the incisors' inclination and position vary in dependance of the sagittal relationship between the upper and lower jaw [28].

Age and gender were considered as possible confounders of cephalometric variables, and hence, subgroup analyses were performed, revealing different measurements indeed. However, this observation must be interpreted with caution, because it might not only be attributed to the confounders age and gender, but also to the small sample size within this group, leading automatically to less variation in the measurements. Detailed subgroup comparisons within each skeletal class (Table 2) revealed that class I children had a more retrognathic mandible and posterior position of the chin compared to adolescents, which can be explained by the anteriorly directed growth and increase in size of the mandible during puberty [26, 29]. Analyzing the sagittal direction in skeletal class II, only comparisons including adult patients resulted in significant differences in sagittal skeletal parameters. Here, in male adults Calculated_ANB was higher, whereas SNB was smaller compared to children and adolescents, which demonstrates the increase in the extent of dysgnathia in adulthood [26]. Eventually, bigger sample sizes would show such differences in female adults too. Differences in the vertical direction were only significant in class I subjects, who presented a more horizontal growth pattern and hypodivergent jaw bases in adolescents compared to children. This could be justified by a decrease in the jaw angle during growth, as also described by Jacob and Buschang [25]. Among the dental variables only few significant differences were found for class I patients, whereas in skeletal class II a higher retroinclination of the upper front teeth was observed in (male) adults compared to younger patients by several parameters (+1/NL, +1/NSL, and +1/NA). This indicates the effect of dysgnathia on dental parameters and describes dental compensation [28], which increases at higher ages, such as the extent of dysgnathia does [26].

### 4.2. Cephalometric Correlations

Statistical analysis revealed several correlations between cephalometric parameters in the same and between different planes for class I and class II patients (Figure 1A). According to our results, in the vertical plane, the divergence of the jaw bases (NL-ML) was positively associated with the inclination of the mandible (ML-NSL) but negatively with the growth pattern (PFH/AFH). This finding demonstrated the effect of the mandible on the vertical skeletal pattern and was also described in other publications: Roy et al. [30] did not specify the type of skeletal malocclusion and used partly slightly different cephalometric parameters, but also found comparable correlations, that is a positive association between the inclination of the mandible (SN-GoGn) and the basal plane angle and a negative one between the basal plane angle and Jarabak ratio (PFH/AFH). In the sagittal direction, a positive correlation was present between the degree of prognathism of the maxilla (SNA) and mandible (SNB) as well as the sagittal position of the chin (SN-Pg). This positive correlation between jaw bases was also found by Segner [12] and demonstrates the need for floating norms in the diagnosis of skeletal class, because even if they do not equal empirical norm values, they still might be harmonious to each other [12]. Furthermore, the correlation between SNB and SN-Pg seems logical, as both parameters are related to the antero-posterior extension of the lower jaw during growth, which is also described in the literature [31, 32]. Considering the sagittal and the vertical direction, the inclination of the mandible (ML-NSL) presented a negative correlation with its sagittal position (SNB) as well as with the sagittal position of the chin (SN-Pg). This finding can be explained by the geometrical relation between those parameters and, regarding SNB and ML-NSL, was also described by Segner [12]. The dental correlations were mainly found between parameters measured in the same jaw and with interincisal angle, which demonstrates that the position and inclination of the incisors did not affect those parameters of the opposite jaw in a relevant way in total class I and II cases.

Considering the influence of age and gender on the correlations (Figure 1B,C), our results indicated that age affects the amount and degree of correlations in both dysgnathia groups, as the most associations were found in adult patients. Whereas gender did not seem to have a relevant effect in class I patients, in class II male adolescent and adult subjects appeared to have more and stronger correlations than females of the same age. However, due to the small sample sizes of the adolescent and adult aged subgroups these observations must be interpreted with caution and require a validation in future studies. Still, a possible explanation for the age-dependent effect might be, that anatomical craniofacial structures influence each other increasingly during growth [15]. For example, Calculated_ANB revealed most correlations in adults, especially in male class II patients. Here, strong positive correlations with Wits appraisal, PFH/AFH and Facial axis and negative associations with growth pattern (ML-NSL, Gonion angle) and divergence of jaw bases (ML-NL) were identified. This illustrates that the antero-posterior relation of the upper and lower jaw, that is skeletal class, depends on many parameters from both the vertical and sagittal direction, which is in agreement with the published floating norms for the ANB angle [12–14, 18].

In summary, the current study highlighted the importance of considering the gender, and age subgroup at the diagnosis, and treatment planning and process. According a study that was performed by Harris [33], on American subjects, and examined the effects of patient age and sex on treatment, found that the age and sex had significant influences on multiple skeletodental variables, and the nature of the correction was affected measurably by the patient's age and sex. In a study that aimed to examine the frequency of orthodontic treatment in German children and adolescents and to examine the influence of age, gender, and socioeconomic status on the frequency of treatment, found that the likelihood of receiving orthodontic treatment was higher for girls (OR = 1.32, 95% CI: 1.06–1.65), for high school pupils (OR = 1.19, 95% CI: 1.06–1.34), and for children and adolescents living in the western part of Germany (OR = 1.45, 95% CI: 1.00–2.08) and increased with age (OR = 1.13, 95% CI: 1.02–1.25) [34]

### 4.3. PCA

The PCA results illustrated that a high percentage of variance (90%) could be explained by the first four principal components, which demonstrates the effectiveness of this method to reduce dimension by keeping the relevant information for the correct diagnosis of skeletal class I and II. This finding is comparable with the results found in classifying skeletal class II and III in the German (91.6% cumulative proportion of the first four components) and Arab population (90% cumulative proportion), which we investigated previously in another study (studies under review). Further analyses revealed that the loadings of those four principal components are affected by skeletal sagittal, skeletal vertical and dental cephalometric parameters. The biggest loading was identified for the fourth principal component, on which ANB had a loading of −0.485. However, analyzing the most relevant variables among all principal components, the inclination of the mandible (ML-NSL) was proven to have the biggest effect, followed by SN-Pg, PFH/AFH and SNB (Figure 2A,B). These results clarify the influence of the mandible in the vertical (ML-NSL) and sagittal (SN-Pg, SNB) direction as well as of the growth pattern (PFH/AFH) on the skeletal class I/II diagnosis and hence the need to consider more parameters than SNA and SNB only. In a previous study that was done by Moreno et al. and examined 309 white Class II adults, PCA results showed that the first seven PCs' explained 81% cumulative variation. The results showed that about half of the variations in the study sample were described by the inclination of the mandibular plane, the angulation of the maxillary incisors, and the mandibular horizontal and vertical lengths [35].

In another study that examined the differences of craniofacial shape in malocclusion by application of two-dimensional geometric morphometrics, found that the first-three PCs were statistically meaningful and explained as 63.24% of total shape variability [36].

### 4.4. Limitations

This study considered only German orthodontic patients to account for differences in cephalometry due to ethnicity. Another limitation might be the heterogeneity of the subgroups, and the fact that the adult subgroup is relatively small, which may cause a limitation in the statistical power. However, we tried to deal with this limitation by using nonparametric tests. Future investigations, however, should aim to match the numbers across different (sub)groups. Besides, in order to prevent selection bias of moderate to severe orthodontic patients, we recommend that future studies include population-based patients, instead of patients from orthodontic clinics.

## 5. Conclusion

Differences in cephalometric parameters between skeletal class I and II demonstrate the need for correct diagnosis of skeletal class, which is affected by other sagittal and vertical variables of the craniofacial skeleton. According to the results of PCA, the mandible's vertical and sagittal position has a strong influence on the diagnosis of skeletal class I/II, which highlights the importance of correctly identifying the corresponding reference marks.

## Figures and Tables

**Table 1:** Cephalometric measurements descriptive statistics.

Variable	M		SD		Min		Pctl. 25		Pctl. 75		Max	
Skeletal class	I	II	I	II	I	II	I	II	I	II	I	II
SNA (°)	81	81	3.8	3.2	63	73	79	79	84	83	92	89
SNB (°)	78	75	3.2	2.9	66	66	76	73	80	77	87	82
SN-Pg (°)	79	76	3.3	3.1	67	67	76	74	81	78	88	85
ANB (°)	3.7	6.3	1.6	1.6	−2.5	1.7	2.7	5.2	4.7	7.4	9	12
Wits appraisal (mm)	0.057	3.6	3.3	5.2	−6	−5	−1.3	1.9	1.7	5.3	8.6	13
ANB individual (°)	3.6	3.5	1.4	1.4	−3.2	−0.5	2.8	2.6	4.6	4.5	7.8	8.1
Calculated_ANB (°)	0.025	2.8	0.84	1.1	−1.5	1.5	−0.68	2	0.78	3.4	1.5	8.6
S-N (mm)	70	72	48	59	42	57	64	63	69	69	81	107
Go-Me (mm)	70	70	47	54	43	54	63	61	70	69	85	100
SN-Ba (°)	132	133	4.9	4.6	117	123	129	130	135	137	144	145
NL/ML (°)	24	23	5.7	6.1	5.3	4	20	19	27	27	46	42
NL/NSL (°)	7.4	8.4	3.6	3.4	−3.1	−1.8	5.1	6.1	9.8	10	18	19
ML/NSL (°)	31	31	6	6.3	16	13	27	27	35	35	52	47
PFH.AFH (%)	67	67	5.1	5.4	50	54	63	63	71	71	80	85
Gonion_Angle (°)	123	119	5.8	6.9	101	104	119	114	126	124	139	139
Facial_Axis (°)	90	89	4.3	4.4	76	71	88	86	93	91	101	99
+1/NL (°)	68	71	8	10	37	46	64	64	73	78	94	113
+1/NSL (°)	76	80	8.4	11	43	53	71	73	81	87	98	122
+1/NA (°)	23	19	7.9	10	2.2	−22	18	12	28	26	54	47
+1/NA (mm)	3.9	2.1	4.2	4.5	−2.4	−12	1.9	0.025	5.3	4.3	11	10
− 1/ML (°)	84	80	6.7	7.3	65	58	79	76	88	84	105	104
− 1/NB (°)	25	26	6.9	7.5	−1.1	4	21	21	30	31	44	41
− 1/NB (mm)	4.2	4.7	4.3	5.6	−3.3	−2.5	2.2	2.6	5.6	5.8	12	12
Interincisal_Angle (°)	128	129	12	14	96	89	120	119	135	135	172	187

*Note:* Cephalometric measurements of patients with skeletal class I and II according to the definition of Panagiotidis and Witt. *M* = mean, SD = standard deviation, Min = minimum, Pctl. 25 = 25% percentile, Pctl. 75 = 75% percentile, Max = maximum.

**Table 2:** Subgroup-differences within skeletal class (I or II).

Parameter	Group comparison	*Z* score	*p*.unadj	*p*.adj
Skeletal class I				
PFH/AFH (%)	I_0 <Age <13–I_14 <age <20	−2.97	0.00	0.01
Gonion angle (°)	I_0 <Age <13–I_14 <age <20	3.04	0.00	0.01
NL/ML (°)	I_0 <Age <13–I_14 <age <20	2.69	0.01	0.02
+1/NSL (°)	I_Female–I_male	2.20	0.03	0.03
+1/NA (°)	I_Female–I_male	−2.57	0.01	0.01
+1/NA (°)	I_0 <Age <13–I_14 <age <20	3.00	0.00	0.01
+1/NA (°)	I_Female_14 <age <20–I_male_0 <age <13	−3.38	0.00	0.01
Skeletal class II				
SNB (°)	II_Female_14 <age <20–II_male_age >21	3.17	0.00	0.02
+1/NL (°)	II_0 <Age <13–II_age >21	−2.43	0.01	0.04
+1/NL (°)	II_Male_0 <age <13–II_male_age >21	−3.18	0.00	0.02
+1/NSL (°)	II_0 <Age <13–II_age >21	−2.50	0.01	0.04
+1/NSL (°)	II_Male_0 <age <13–II_male_age >21	−3.56	0.00	0.01
+1/NA (°)	II_0 <Age <13–II_age >21	2.43	0.02	0.045
+1/NA (°)	II_Male_0 <Age <13–II_male_age >21	3.17	0.00	0.02

*Note:* Subgroup differences within skeletal class I and II patients with statistical difference according to Kruskal–Wallis with post hoc Dunn analysis and Bonferroni correction. The post hoc test was done separately for each skeletal class. Statistical significance was set at *p* < 0.01 and *p* < 0.05. *Z* = difference between the mean ranks of each two groups, *p*.unadj = unadjusted *p*-value in Dunn's test, *p*.adj = adjusted *p*-value (Bonferroni).

**Table 3:** Significant differences in skeletal parameters between different skeletal classes.

Parameter	Groups	*Z* score	*p*.unadj	*p*.adj
Calculated_ANB	I_Female–II_female	−14.62	0.00	0.00
Calculated_ANB	I_Male–II_male	−13.33	0.00	0.00
Calculated_ANB	I_0 <Age <13–II_0 <age <13	−17.30	0.00	0.00
Calculated_ANB	I_14 <Age <20–II_14 <age <20	−8.14	0.00	0.00
Calculated_ANB	I_Age >21–II_age >21	−5.14	0.00	0.00
Calculated_ANB	I_Female_age >21–II_male_age >21	−3.93	0.00	0.01
Calculated_ANB	I_Male_age >21–II_male_age >21	−3.80	0.00	0.01
Wits	I_Female–II_female	−10.33	0.00	0.00
Wits	I_Male–II_male	−9.03	0.00	0.00
Wits	I_0 <Age <13–II_0 <age <13	−12.32	0.00	0.00
Wits	I_14 <Age <20–II_14 <age <20	−4.55	0.00	0.00
Wits	I_Age >21–II_age >21	−3.69	0.00	0.00
Wits	I_Female_0 <age <13–II_male_age >21	−3.95	0.00	0.01
Wits	I_Male_0 <age <13–II_male_age >21	−3.50	0.00	0.03
SNB	I_Female–II_female	7.08	0.00	0.00
SNB	I_Male–II_male	7.21	0.00	0.00
SNB	I_0 <Age <13–II_0 <age <13	8.41	0.00	0.00
SNB	I_14 <Age <20–II_14 <age <20	4.46	0.00	0.00
SNB	I_Age >21–II_age >21	3.29	0.00	0.01
SNB	I_Female_14 <age <20–II_male_age >21	4.65	0.00	0.00
SNB	I_Male_14 <age <20–II_male_age >21	4.36	0.00	0.00
SNPg	I_Female–II_female	6.28	0.00	0.00
SNPg	I_Male–II_male	6.16	0.00	0.00
SNPg	I_0 <Age <13–II_0 <age <13	7.40	0.00	0.00
SNPg	I_14 <Age <20–II_0 <age <13	7.21	0.00	0.00
SNPg	I_Female_14 <age <20–II_male_age >21	3.55	0.00	0.03
NL-NSL	I_Male–II_male	−3.55	0.00	0.00
Gonion angle	I_Female–II_female	4.80	0.00	0.00
Gonion angle	I_Male–II_male	3.40	0.00	0.00
Gonion angle	I_0 <Age <13–II_0 <age <13	5.15	0.00	0.00
Gonion angle	I_Female_0 <age <13–II_female_0 <age <13	4.07	0.00	0.00
Gonion angle	I_Male_0 <age <13–II_female_age >21	3.44	0.00	0.04
Facial axis	I_Female–II_female	3.45	0.00	0.00
Facial axis	I_0 <Age <13–II_0 <age <13	3.81	0.00	0.00
Facial axis	I_Male_0 <age <13–II_female_0 <age <13	3.89	0.00	0.01

*Note:* Significant differences in skeletal parameters between different skeletal classes, considering age and gender subgroups, according to Kruskal–Wallis with post hoc Dunn analysis and Bonferroni correction. Statistical significance was set at *p* < 0.01 and *p* < 0.05. *Z* = difference between the mean ranks of each two groups, *p*.unadj = unadjusted *p*-value in Dunn's test, *p*.adj = adjusted *p*-value (Bonferroni).

**Table 4:** Significant variations in dental parameters between different skeletal classes.

Parameter	Groups	*Z* score	*p*.unadj	*p*.adj
+1/NL	I_Female–II_female	−2.73	0.01	0.04
+1/NL	I_Age >21–II_age >21	−3.25	0.00	0.02
+1/NL	I_Male_0 <age <13–II_male_age >21	−3.45	0.00	0.04
+1/NL	I_Male_age >21–II_male_age >21	−3.51	0.00	0.03
+1/NA (mm)	I_Female–II_female	4.31	0.00	0.00
+1/NA (mm)	I_Male–II_male	4.40	0.00	0.00
+1/NA (mm)	I_0 <Age <13–II_0 <age <13	5.03	0.00	0.00
+1/NA (mm)	I_Age >21–II_age >21	3.44	0.00	0.01
+1/NA (mm)	I_Male_0 <age <13–II_male_age >21	3.46	0.00	0.04
− 1/ML	I_Female–II_female	4.29	0.00	0.00
− 1/ML	I_Male–II_male	3.71	0.00	0.00
− 1/ML	I_0 <Age <13–II_0 <age <13	4.74	0.00	0.00
− 1/ML	I_14 <Age <20–II_14 <age <20	3.22	0.00	0.02
− 1/ML	I_Female_0 <age <13–II_female_14 <age <20	3.84	0.00	0.01
−1/ML	I_Male_0 <age <13–II_female_14 <age <20	4.30	0.00	0.00

*Note:* Significant differences in dental parameters between different skeletal classes, considering age and gender subgroups, according to Kruskal–Wallis with post hoc Dunn analysis and Bonferroni correction. Statistical significance was set at *p* < 0.01 and *p* < 0.05. *Z* = difference between the mean ranks of each two groups, *p*.unadj = unadjusted *p*-value in Dunn's test, *p*.adj = adjusted *p*-value (Bonferroni).

**Table 5:** The four most relevant principal components.

Most relevant principle components	PC 1	PC 2	PC 3	PC 4
Standard deviation	1.19	0.98	0.61	0.54
Proportion of variance	0.42	0.28	0.11	0.08
Cumulative proportion	0.42	0.70	0.81	0.90

*Note:* The four most relevant principal components to classify an individual correctly as skeletal class I or II.

Abbreviation: PC, principal component.

**Table 6:** Loadings of the four most relevant principal components.

Cephalometric parameter	PC 1	PC 2	PC 3	PC 4
NL-ML angle	0.26	0.16	0.21	0.03
NL-NSL	0.20	−0.03	−0.10	0.02
PFH/AFH	−0.36	−0.13	−0.15	−0.02
Gonial angle	0.20	0.16	0.28	−0.01
Facial axis	−0.29	−0.04	0.05	−0.06
SNA angle	−0.28	0.00	−0.07	0.18
SNB angle	−0.37	0.00	0.11	0.01
ANB angle	0.12	0.00	−0.31	0.29
Calculated_ANB	0.12	−0.10	−0.43	0.18
SN-Ba angle	0.17	0.04	−0.03	0.07
SN-Pg angle	−0.38	−0.05	0.10	−0.03
S-N (mm)	0.02	0.13	−0.23	−0.48
Go-Me (mm)	0.01	0.12	−0.23	−0.48
Wits appraisal (mm)	0.03	−0.04	−0.36	−0.09
ML-NSL angle	0.37	0.13	0.14	0.04
+1/NL angle	0.13	−0.37	−0.07	−0.09
+1/SNL angle	0.20	−0.36	−0.10	−0.08
+1/NA angle	−0.10	0.38	0.13	0.02
+1/NA (mm)	−0.05	0.35	−0.06	−0.32
−1/ML (anatomic)	0.12	−0.15	0.36	−0.26
−1/NB angle	0.02	0.27	−0.19	0.30
−1/NB (mm)	0.06	0.26	−0.27	−0.22
Interincisal angle	0.04	−0.42	0.06	−0.23

*Note:* Loadings of the four most relevant principal components to classify an individual correctly as skeletal class I or II. Loadings were highlighted if the value was ≤ −0.300 or ≥ 0.300.

Abbreviation: PC, principal component.

## Data Availability

Data used in this study will be available from the corresponding author upon request.

## Supporting Information

Supporting Information Table S1: List and definition of the relevant skeletal and dental cephalometric variables analyzed. Table S2: Demographic characteristics of the study groups and distribution to the subgroups, *n* = absolute numbers, % = relative frequency. Table S3: Significant differences in vertical skeletal parameters between different skeletal classes, considering age and gender subgroups, according to Kruskal–Wallis with post hoc Dunn analysis and Bonferroni correction. Statistical significance was set at *p* < 0.01 and *p* < 0.05. *Z* = difference between the mean ranks of each two groups, *p*.unadj = unadjusted *p*-value in Dunn's test, *p*.adj = adjusted *p*-value (Bonferroni). Table S4: Significant differences in sagittal skeletal parameters between different skeletal classes, considering age and gender subgroups, according to Kruskal–Wallis with post hoc Dunn analysis and Bonferroni correction. Statistical significance was set at *p* < 0.01 and *p* < 0.05. *Z* = difference between the mean ranks of each two groups, *p*.unadj = unadjusted *p*-value in Dunn's test, *p*.adj = adjusted *p*-value (Bonferroni). Table S5: Significant differences in dental parameters between different skeletal classes, considering age and gender subgroups, according to Kruskal–Wallis with post hoc Dunn analysis and Bonferroni correction. Statistical significance was set at *p* < 0.01 and p < 0.05. *Z* = difference between the mean ranks of each two groups, *p*.unadj = unadjusted *p*-value in Dunn's test, *p*.adj = adjusted *p*-value (Bonferroni). Table S6: Significant differences in calculated_ANB between different skeletal classes, considering age and gender subgroups, according to Kruskal–Wallis with post hoc Dunn analysis and Bonferroni correction. Statistical significance was set at *p* < 0.01 and *p* < 0.05. *Z* = difference between the mean ranks of each two groups, *p*.unadj = unadjusted *p*-value in Dunn's test, *p*.adj = adjusted *p*-value (Bonferroni). Table S7: Subgroup differences within skeletal class II and I patients with a statistical difference according to Kruskal–Wallis with post hoc Dunn analysis and Bonferroni correction. The post hoc test was done separately for each skeletal class. Statistical significance was set at *p* < 0.01 and *p* < 0.05. *Z* = difference between the mean ranks of each two groups, *p*.unadj = unadjusted *p*-value in Dunn's test, *p*.adj = adjusted *p*-value (Bonferroni). Figure S1: Reference lines and marks needed for cephalometric evaluation. Angle 1 = facial axis. Details are described in Table 1.
