# Supplementary material for: Cross-Sectional Observational Study of the Differences in Cephalometric Parameters in German Class I/II Orthodontic Patients
Source: Int J Dent. 2025 Aug 21;2025:9665260. doi: 10.1155/ijod/9665260 (PMC12393958; doi:10.1155/ijod/9665260)
Supplement: Supporting Information — Table S1: List and definition of the relevant skeletal and dental cephalometric variables analyzed. Table S2: Demographic characteristics of the study groups and distribution to the subgroups, n = absolute numbers, % = relative frequency. Table S3: Significant differences in vertical skeletal parameters between different skeletal classes, considering age and gender subgroups, according to Kruskal–Wallis with post hoc Dunn analysis and Bonferroni correction. Statistical significance was set at p < 0.01 and p < 0.05. Z = difference between the mean ranks of each two groups, p.unadj = unadjusted p-value in Dunn's test, p.adj = adjusted p-value (Bonferroni). Table S4: Significant differences in sagittal skeletal parameters between different skeletal classes, considering age and gender subgroups, according to Kruskal–Wallis with post hoc Dunn analysis and Bonferroni correction. Statistical significance was set at p < 0.01 and p < 0.05. Z = difference between the mean ranks of each two groups, p.unadj = unadjusted p-value in Dunn's test, p.adj = adjusted p-value (Bonferroni). Table S5: Significant differences in dental parameters between different skeletal classes, considering age and gender subgroups, according to Kruskal–Wallis with post hoc Dunn analysis and Bonferroni correction. Statistical significance was set at p < 0.01 and p < 0.05. Z = difference between the mean ranks of each two groups, p.unadj = unadjusted p-value in Dunn's test, p.adj = adjusted p-value (Bonferroni). Table S6: Significant differences in calculated_ANB between different skeletal classes, considering age and gender subgroups, according to Kruskal–Wallis with post hoc Dunn analysis and Bonferroni correction. Statistical significance was set at p < 0.01 and p < 0.05. Z = difference between the mean ranks of each two groups, p.unadj = unadjusted p-value in Dunn's test, p.adj = adjusted p-value (Bonferroni). Table S7: Subgroup differences within skeletal class II and I patients with a statistical [file 9665260.f1.docx]

**Supplementary Material**

**S. Table 1:** Cephalometric parameters definitions

| **variable** | **definition** |
| --- | --- |
| **skeletal sagittal** | |
| SNA [°] | caudal angle between Sella (S), Nasion (N) and point A (A) |
| SNB [°] | caudal angle between Sella, Nasion and point B (B) |
| ANB [°] | caudal angle between Nasion, point A and point B) |
| Wits appraisal [mm] | anterior-posterior distance between perpendiculars through A (A’) and B (B’) at occlusal plane (Occl)  < 0:B’ is anterior to A’  ≥ 0: B’ is posterior to A’ |
| SN-Ba [°] | caudal angle between Sella, Nasion and Basion (Ba) |
| SN-Pg [°] | caudal angle between Sella, Nasion and Pogonion (Pg) |
| S-N [mm] | distance between Sella and Nasion, length of the anterior cranial base |
| Go-Me [mm] | distance between Gonion (Go) and Menton (Me), length of mandibular plane (ML) |
| **skeletal vertical/ growth** | |
| NL/NSL | anterior angle between Sella-Nasion-line (NSL) and nasal line (NL) |
| ML/NSL | anterior angle between NSL and ML |
| NL/ML | anterior angle between the lines NL and ML |
| PFH/AFH | ratio between posterior (S-Go, PFH) and anterior (N-Me, AFH) facial height; growth pattern |
| Gonion angle | anterior angle between ML and line Go-Ar; growth pattern |
| Facial axis | posterior-caudal angle between the lines N-Ba and Pt-GN’; growth pattern |
| **dental** | |
| +1/NL [°] | anterior-caudal angle between upper incisors’ tooth axis (+1) and NL |
| +1/NSL [°] | anterior-caudal angle between +1 and NSL |
| +1/NA [°] | caudal angle between upper incisors’ tooth axis and line NA |
| +1/NA [mm] | perpendicular distance between line NA and upper incisal point (+1i) |
| -1/ML [°] | anterior-cranial angle between lower incisors’ tooth axis and line ML |
| -1/NB [°] | caudal angle between lower incisors’ tooth axis and line NB |
| -1/NB [mm] | perpendicular distance between NB and lower incisal point (-1i) |
| Interincisal_angle [°] | posterior angle between +1 and -1 |

**S. Table *1*:** List and definition of the relevant skeletal and dental cephalometric variables analysed.

**S. Table 2:** Demographic characteristics of the study groups

| group | (n, %) | age (years) | age: ≤ 13  (n, %) | age: 14-21  (n, %) | age: > 21  (n, %) | gender | | |
| --- | --- | --- | --- | --- | --- | --- | --- | --- |
|  |  |  |  |  |  | female (n, %) | male (n, %) |  |
| class I | 346, 62 | 13 ± 4 | 275, 79 | 57, 16 | 14, 4 | 194, 56 | 152, 44 |  |
| class I female | - | - | 154, 45 | 32, 9 | 8, 2 | - | - |  |
| class I male | - | - | 121, 35 | 25, 7 | 6, 2 | - | - |  |
| class II | 210, 38 | 13 ± 6.6 | 164, 78 | 33, 16 | 13, 6 | 125, 60 | 85, 40 |  |
| class II female | - | - | 96, 46 | 22, 10 | 7, 3 | - | - |  |
| class II male | - | - | 68, 32 | 11, 5 | 6, 3 | - | - |  |

**S. Table 2:** Demographic characteristics of the study groups and distribution to the subgroups. n = absolute numbers, % = relative frequency.

**S. Table 3**: Significant differences in vertical skeletal parameters between different skeletal classes

| Parameter | groups | z-score | un-adj | adj |
| --- | --- | --- | --- | --- |
| NL/NSL | I_Male - II_Female | -2.81 | 0.00 | 0.03 |
| PFH/AFH | I_0<Age<13 - I_14<Age<20 | -2.96 | 0.00 | 0.05 |
| Gonial angle | I_Male - II_Female | 5.30 | 0.00 | 0.00 |
| Gonial angle | I_Female - II_Male | 2.86 | 0.00 | 0.03 |
| Gonial angle | I_0<Age<13 - II_14<Age<20 | 4.72 | 0.00 | 0.00 |
| Gonial angle | I_0<Age<13 - II_Age>21 | 3.17 | 0.00 | 0.02 |
| Gonial angle | I_Male_0<Age<13 - II_Female_0<Age<13 | 4.61 | 0.00 | 0.00 |
| Gonial angle | I_Male_0<Age<13 - II_Female_14<Age<20 | 3.89 | 0.00 | 0.01 |
| Gonial angle | I_Female_0<Age<13 - II_Female_14<Age<20 | 3.51 | 0.00 | 0.03 |
| Facial axis | I_Male - II_Female | 4.15 | 0.00 | 0.00 |
| Facial axis | I_Male - II_Male | 2.76 | 0.01 | 0.03 |
| Facial axis | I_14<Age<20 - II_0<Age<13 | 3.22 | 0.00 | 0.02 |

**S. Table 3**: Significant differences in vertical skeletal parameters between different skeletal classes, considering age and gender subgroups, according to Kruskal Wallis with post hoc Dunn analysis and Bonferroni correction. Statistical significance was set at p < 0.01 and p < 0.05. Z = difference between the mean ranks of each two groups, p.unadj = unadjusted p-value in Dunn's test, p adj = adjusted p-value (Bonferroni).

**S. Table 4:** Significant differences in sagittal skeletal parameters between different skeletal classes

| Parameter | groups | z | un-adj | adj |
| --- | --- | --- | --- | --- |
| SNB angle | I_Female - II_Male | 7.17 | 0.00 | 0.00 |
| SNB angle | I_Male - II_Female | 7.09 | 0.00 | 0.00 |
| SNB angle | I_14<Age<20 - II_0<Age<13 | 7.53 | 0.00 | 0.00 |
| SNB angle | I_14<Age<20 - II_Age>21 | 4.91 | 0.00 | 0.00 |
| SNB angle | I_0<Age<13 - II_Age>21 | 4.16 | 0.00 | 0.00 |
| SNB angle | I_0<Age<13 - II_14<Age<20 | 3.51 | 0.00 | 0.01 |
| SNB angle | I_Age>21 - II_0<Age<13 | 3.30 | 0.00 | 0.01 |
| SNB angle | I_Male_0<Age<13 - II_Female_0<Age<13 | 6.34 | 0.00 | 0.00 |
| SNB angle | I_Female_0<Age<13 - II_Female_0<Age<13 | 6.06 | 0.00 | 0.00 |
| SNB angle | I_Male_0<Age<13 - II_Male_0<Age<13 | 5.82 | 0.00 | 0.00 |
| SNB angle | I_Female_14<Age<20 - II_Female_0<Age<13 | 5.82 | 0.00 | 0.00 |
| SNB angle | I_Female_14<Age<20 - II_Male_0<Age<13 | 5.62 | 0.00 | 0.00 |
| SNB angle | I_Female_0<Age<13 - II_Male_0<Age<13 | 5.53 | 0.00 | 0.00 |
| SNB angle | I_Male_14<Age<20 - II_Female_0<Age<13 | 4.91 | 0.00 | 0.00 |
| SNB angle | I_Male_14<Age<20 - II_Male_0<Age<13 | 4.78 | 0.00 | 0.00 |
| SNB angle | I_Male_0<Age<13 - II_Male_Age>21 | 4.18 | 0.00 | 0.00 |
| SNB angle | I_Female_0<Age<13 - II_Male_Age>21 | 4.01 | 0.00 | 0.00 |
| SNB angle | I_Female_14<Age<20 - II_Female_14<Age<20 | 3.39 | 0.00 | 0.05 |
| SNB angle | I_Female_Age>21 - II_Male_Age>21 | 3.38 | 0.00 | 0.05 |
| ANB angle | I_Female - II_Female | -11.32 | 0.00 | 0.00 |
| ANB angle | I_Female - II_Male | -10.67 | 0.00 | 0.00 |
| ANB angle | I_Male - II_Female | -10.53 | 0.00 | 0.00 |
| ANB angle | I_Male - II_Male | -10.05 | 0.00 | 0.00 |
| ANB angle | I_0<Age<13 - II_0<Age<13 | -13.18 | 0.00 | 0.00 |
| ANB angle | I_14<Age<20 - II_0<Age<13 | -8.69 | 0.00 | 0.00 |
| ANB angle | I_0<Age<13 - II_14<Age<20 | -7.16 | 0.00 | 0.00 |
| ANB angle | I_14<Age<20 - II_14<Age<20 | -6.19 | 0.00 | 0.00 |
| ANB angle | I_Age>21 - II_0<Age<13 | -5.47 | 0.00 | 0.00 |
| ANB angle | I_0<Age<13 - II_Age>21 | -4.84 | 0.00 | 0.00 |
| ANB angle | I_Age>21 - II_14<Age<20 | -4.83 | 0.00 | 0.00 |
| ANB angle | I_14<Age<20 - II_Age>21 | -4.59 | 0.00 | 0.00 |
| ANB angle | I_Age>21 - II_Age>21 | -4.14 | 0.00 | 0.00 |
| ANB angle | I_Female_0<Age<13 - II_Female_0<Age<13 | -9.80 | 0.00 | 0.00 |
| ANB angle | I_Male_0<Age<13 - II_Female_0<Age<13 | -9.43 | 0.00 | 0.00 |
| ANB angle | I_Female_0<Age<13 - II_Male_0<Age<13 | -9.08 | 0.00 | 0.00 |
| ANB angle | I_Male_0<Age<13 - II_Male_0<Age<13 | -8.81 | 0.00 | 0.00 |
| ANB angle | I_Female_14<Age<20 - II_Female_0<Age<13 | -6.85 | 0.00 | 0.00 |
| ANB angle | I_Female_14<Age<20 - II_Male_0<Age<13 | -6.74 | 0.00 | 0.00 |
| ANB angle | I_Female_0<Age<13 - II_Female_14<Age<20 | -5.57 | 0.00 | 0.00 |
| ANB angle | I_Male_0<Age<13 - II_Female_14<Age<20 | -5.53 | 0.00 | 0.00 |
| ANB angle | I_Male_14<Age<20 - II_Female_0<Age<13 | -5.41 | 0.00 | 0.00 |
| ANB angle | I_Male_14<Age<20 - II_Male_0<Age<13 | -5.39 | 0.00 | 0.00 |
| ANB angle | I_Female_14<Age<20 - II_Female_14<Age<20 | -5.02 | 0.00 | 0.00 |
| ANB angle | I_Male_0<Age<13 - II_Male_14<Age<20 | -4.49 | 0.00 | 0.00 |
| ANB angle | I_Female_0<Age<13 - II_Male_14<Age<20 | -4.48 | 0.00 | 0.00 |
| ANB angle | I_Female_14<Age<20 - II_Male_14<Age<20 | -4.36 | 0.00 | 0.00 |
| ANB angle | I_Female_Age>21 - II_Male_0<Age<13 | -4.33 | 0.00 | 0.00 |
| ANB angle | I_Female_Age>21 - II_Female_0<Age<13 | -4.27 | 0.00 | 0.00 |
| ANB angle | I_Male_14<Age<20 - II_Female_14<Age<20 | -4.13 | 0.00 | 0.00 |
| ANB angle | I_Female_14<Age<20 - II_Male_Age>21 | -3.98 | 0.00 | 0.00 |
| ANB angle | I_Male_0<Age<13 - II_Male_Age>21 | -3.98 | 0.00 | 0.00 |
| ANB angle | I_Female_0<Age<13 - II_Male_Age>21 | -3.96 | 0.00 | 0.00 |
| ANB angle | I_Female_Age>21 - II_Female_14<Age<20 | -3.79 | 0.00 | 0.01 |
| ANB angle | I_Male_14<Age<20 - II_Male_14<Age<20 | -3.70 | 0.00 | 0.01 |
| ANB angle | I_Female_Age>21 - II_Male_14<Age<20 | -3.65 | 0.00 | 0.02 |
| ANB angle | I_Female_Age>21 - II_Male_Age>21 | -3.61 | 0.00 | 0.02 |
| ANB angle | I_Male_14<Age<20 - II_Male_Age>21 | -3.49 | 0.00 | 0.03 |
| ANB angle | I_Male_Age>21 - II_Male_0<Age<13 | -3.42 | 0.00 | 0.04 |
| SNPg angle | I_Male - II_Female | 6.68 | 0.00 | 0.00 |
| SNPg angle | I_Female - II_Male | 5.75 | 0.00 | 0.00 |
| SNPg angle | I_14<Age<20 - II_14<Age<20 | 4.01 | 0.00 | 0.00 |
| SNPg angle | I_14<Age<20 - II_Age>21 | 3.96 | 0.00 | 0.00 |
| SNPg angle | I_Age>21 - II_0<Age<13 | 3.22 | 0.00 | 0.02 |
| SNPg angle | I_0<Age<13 - II_Age>21 | 2.96 | 0.00 | 0.05 |
| SNPg angle | I_Male_0<Age<13 - II_Female_0<Age<13 | 5.97 | 0.00 | 0.00 |
| SNPg angle | I_Female_14<Age<20 - II_Female_0<Age<13 | 5.63 | 0.00 | 0.00 |
| SNPg angle | I_Female_0<Age<13 - II_Female_0<Age<13 | 5.36 | 0.00 | 0.00 |
| SNPg angle | I_Female_14<Age<20 - II_Male_0<Age<13 | 5.14 | 0.00 | 0.00 |
| SNPg angle | I_Male_0<Age<13 - II_Male_0<Age<13 | 5.07 | 0.00 | 0.00 |
| SNPg angle | I_Male_14<Age<20 - II_Female_0<Age<13 | 4.90 | 0.00 | 0.00 |
| SNPg angle | I_Male_14<Age<20 - II_Male_0<Age<13 | 4.50 | 0.00 | 0.00 |
| SNPg angle | I_Female_0<Age<13 - II_Male_0<Age<13 | 4.46 | 0.00 | 0.00 |
| S-N (mm) | I_Female - I_Male | -4.22 | 0.00 | 0.00 |
| S-N (mm) | I_Male - II_Female | 3.94 | 0.00 | 0.00 |
| S-N (mm) | I_14<Age<20 - II_0<Age<13 | 4.19 | 0.00 | 0.00 |
| S-N (mm) | II_0<Age<13 - II_Age>21 | -4.10 | 0.00 | 0.00 |
| S-N (mm) | I_0<Age<13 - II_Age>21 | -3.52 | 0.00 | 0.01 |
| S-N (mm) | I_0<Age<13 - I_14<Age<20 | -3.17 | 0.00 | 0.02 |
| S-N (mm) | II_0<Age<13 - II_14<Age<20 | -2.95 | 0.00 | 0.05 |
| S-N (mm) | I_Male_14<Age<20 - II_Female_0<Age<13 | 5.18 | 0.00 | 0.00 |
| S-N (mm) | I_Female_0<Age<13 - I_Male_14<Age<20 | -4.86 | 0.00 | 0.00 |
| S-N (mm) | I_Male_0<Age<13 - II_Female_0<Age<13 | 4.08 | 0.00 | 0.00 |
| S-N (mm) | II_Female_0<Age<13 - II_Male_Age>21 | -3.79 | 0.00 | 0.01 |
| S-N (mm) | I_Male_14<Age<20 - II_Male_0<Age<13 | 3.66 | 0.00 | 0.02 |
| S-N (mm) | I_Female_0<Age<13 - I_Male_0<Age<13 | -3.64 | 0.00 | 0.02 |
| S-N (mm) | I_Female_0<Age<13 - II_Male_Age>21 | -3.56 | 0.00 | 0.02 |
| Go-Me (mm) | I_Male - II_Female | 4.94 | 0.00 | 0.00 |
| Go-Me (mm) | I_Male - II_Male | 3.09 | 0.00 | 0.01 |
| Go-Me (mm) | I_Female - I_Male | -2.94 | 0.00 | 0.02 |
| Go-Me (mm) | I_14<Age<20 - II_0<Age<13 | 8.27 | 0.00 | 0.00 |
| Go-Me (mm) | I_0<Age<13 - I_14<Age<20 | -6.05 | 0.00 | 0.00 |
| Go-Me (mm) | II_0<Age<13 - II_Age>21 | -5.62 | 0.00 | 0.00 |
| Go-Me (mm) | I_0<Age<13 - II_Age>21 | -4.33 | 0.00 | 0.00 |
| Go-Me (mm) | I_0<Age<13 - II_0<Age<13 | 3.96 | 0.00 | 0.00 |
| Go-Me (mm) | II_0<Age<13 - II_14<Age<20 | -3.30 | 0.00 | 0.01 |
| Go-Me (mm) | II_14<Age<20 - II_Age>21 | -3.03 | 0.00 | 0.04 |
| Go-Me (mm) | I_14<Age<20 - II_14<Age<20 | 2.94 | 0.00 | 0.05 |
| Go-Me (mm) | I_Male_14<Age<20 - II_Female_0<Age<13 | 7.23 | 0.00 | 0.00 |
| Go-Me (mm) | I_Female_0<Age<13 - I_Male_14<Age<20 | -5.95 | 0.00 | 0.00 |
| Go-Me (mm) | I_Male_14<Age<20 - II_Male_0<Age<13 | 5.95 | 0.00 | 0.00 |
| Go-Me (mm) | I_Female_14<Age<20 - II_Female_0<Age<13 | 5.72 | 0.00 | 0.00 |
| Go-Me (mm) | I_Male_0<Age<13 - II_Female_0<Age<13 | 4.93 | 0.00 | 0.00 |
| Go-Me (mm) | II_Female_0<Age<13 - II_Female_Age>21 | -4.50 | 0.00 | 0.00 |
| Go-Me (mm) | I_Female_14<Age<20 - II_Male_0<Age<13 | 4.37 | 0.00 | 0.00 |
| Go-Me (mm) | I_Male_0<Age<13 - I_Male_14<Age<20 | -4.32 | 0.00 | 0.00 |
| Go-Me (mm) | I_Female_0<Age<13 - I_Female_14<Age<20 | -4.26 | 0.00 | 0.00 |
| Go-Me (mm) | II_Female_0<Age<13 - II_Male_Age>21 | -3.96 | 0.00 | 0.01 |
| Go-Me (mm) | II_Female_Age>21 - II_Male_0<Age<13 | 3.85 | 0.00 | 0.01 |
| Go-Me (mm) | I_Female_0<Age<13 - II_Female_Age>21 | -3.67 | 0.00 | 0.02 |
| Wits appraisal | I_Female - II_Male | -10.99 | 0.00 | 0.00 |
| Wits appraisal | I_Male - II_Female | -8.11 | 0.00 | 0.00 |
| Wits appraisal | I_0<Age<13 - II_14<Age<20 | -6.97 | 0.00 | 0.00 |
| Wits appraisal | I_14<Age<20 - II_0<Age<13 | -6.03 | 0.00 | 0.00 |
| Wits appraisal | I_0<Age<13 - II_Age>21 | -5.37 | 0.00 | 0.00 |
| Wits appraisal | I_14<Age<20 - II_Age>21 | -4.02 | 0.00 | 0.00 |
| Wits appraisal | I_Age>21 - II_0<Age<13 | -4.00 | 0.00 | 0.00 |
| Wits appraisal | I_Age>21 - II_14<Age<20 | -3.71 | 0.00 | 0.00 |
| Wits appraisal | I_Female_0<Age<13 - II_Male_0<Age<13 | -10.06 | 0.00 | 0.00 |
| Wits appraisal | I_Female_0<Age<13 - II_Female_0<Age<13 | -9.02 | 0.00 | 0.00 |
| Wits appraisal | I_Male_0<Age<13 - II_Male_0<Age<13 | -8.48 | 0.00 | 0.00 |
| Wits appraisal | I_Male_0<Age<13 - II_Female_0<Age<13 | -7.27 | 0.00 | 0.00 |
| Wits appraisal | I_Female_0<Age<13 - II_Female_14<Age<20 | -5.90 | 0.00 | 0.00 |
| Wits appraisal | I_Female_14<Age<20 - II_Male_0<Age<13 | -5.82 | 0.00 | 0.00 |
| Wits appraisal | I_Male_0<Age<13 - II_Female_14<Age<20 | -5.03 | 0.00 | 0.00 |
| Wits appraisal | I_Female_14<Age<20 - II_Female_0<Age<13 | -4.68 | 0.00 | 0.00 |
| Wits appraisal | I_Female_0<Age<13 - II_Male_14<Age<20 | -4.49 | 0.00 | 0.00 |
| Wits appraisal | I_Female_14<Age<20 - II_Female_14<Age<20 | -4.07 | 0.00 | 0.00 |
| Wits appraisal | I_Female_0<Age<13 - II_Female_Age>21 | -4.05 | 0.00 | 0.00 |
| Wits appraisal | I_Male_0<Age<13 - II_Male_14<Age<20 | -3.88 | 0.00 | 0.01 |
| Wits appraisal | I_Male_14<Age<20 - II_Male_0<Age<13 | -3.87 | 0.00 | 0.01 |
| Wits appraisal | I_Female_Age>21 - II_Male_0<Age<13 | -3.63 | 0.00 | 0.02 |
| Wits appraisal | I_Male_0<Age<13 - II_Female_Age>21 | -3.57 | 0.00 | 0.02 |
| Wits appraisal | I_Female_14<Age<20 - II_Male_14<Age<20 | -3.38 | 0.00 | 0.05 |

**S. Table 4:** Significant differences in sagittal skeletal parameters between different skeletal classes, considering age and gender subgroups, according to Kruskal Wallis with post hoc Dunn analysis and Bonferroni correction. Statistical significance was set at p < 0.01 and p < 0.05. Z = difference between the mean ranks of each two groups, p.unadj = unadjusted p-value in Dunn's test, p adj = adjusted p-value (Bonferroni).

**S. Table 5:** Significant differences in dental parameters between different skeletal classes

| **parameter** | **Group comparison** | **Z-score** | **P. unadj** | **p adj** |
| --- | --- | --- | --- | --- |
| +1/NL angle | I_Male - II_Female | -3.90 | 0.00 | 0.00 |
| +1/NL angle | I_0<Age<13 - II_Age>21 | -3.39 | 0.00 | 0.01 |
| +1/NSL angle | I_Male - II_Female | -5.00 | 0.00 | 0.00 |
| +1/NSL angle | I_Female - II_Female | -3.35 | 0.00 | 0.00 |
| +1/NSL angle | I_Male - II_Male | -2.74 | 0.01 | 0.04 |
| +1/NSL angle | I_0<Age<13 - II_Age>21 | -3.76 | 0.00 | 0.00 |
| +1/NSL angle | I_Age>21 - II_Age>21 | -3.59 | 0.00 | 0.00 |
| +1/NSL angle | I_0<Age<13 - II_0<Age<13 | -3.45 | 0.00 | 0.01 |
| +1/NSL angle | I_Male_0<Age<13 - II_Female_0<Age<13 | -4.46 | 0.00 | 0.00 |
| +1/NSL angle | I_Male_0<Age<13 - II_Male_Age>21 | -4.16 | 0.00 | 0.00 |
| +1/NSL angle | I_Male_Age>21 - II_Male_Age>21 | -3.85 | 0.00 | 0.01 |
| +1/NSL angle | I_Female_0<Age<13 - II_Male_Age>21 | -3.63 | 0.00 | 0.02 |
| +1/NSL angle | II_Male_0<Age<13 - II_Male_Age>21 | -3.45 | 0.00 | 0.04 |
| +1/NA angle | I_Male - II_Female | 4.96 | 0.00 | 0.00 |
| +1/NA angle | I_Female - II_Female | 3.02 | 0.00 | 0.02 |
| +1/NA angle | I_Male - II_Male | 2.81 | 0.00 | 0.03 |
| +1/NA angle | I_0<Age<13 - II_Age>21 | 3.64 | 0.00 | 0.00 |
| +1/NA angle | I_0<Age<13 - II_0<Age<13 | 3.50 | 0.00 | 0.01 |
| +1/NA angle | I_Age>21 - II_Age>21 | 3.33 | 0.00 | 0.01 |
| +1/NA angle | I_0<Age<13 - II_14<Age<20 | 3.21 | 0.00 | 0.02 |
| +1/NA angle | I_Male_0<Age<13 - II_Female_0<Age<13 | 4.56 | 0.00 | 0.00 |
| +1/NA angle | I_Male_0<Age<13 - II_Male_Age>21 | 3.93 | 0.00 | 0.01 |
| +1/NA angle | I_Male_0<Age<13 - II_Female_14<Age<20 | 3.49 | 0.00 | 0.03 |
| +1/NA angle | I_Male_Age>21 - II_Male_Age>21 | 3.43 | 0.00 | 0.04 |
| +1/NA (mm) | I_Male - II_Female | 5.83 | 0.00 | 0.00 |
| +1/NA (mm) | I_Female - II_Male | 2.98 | 0.00 | 0.02 |
| +1/NA (mm) | I_0<Age<13 - II_14<Age<20 | 3.59 | 0.00 | 0.00 |
| +1/NA (mm) | I_Age>21 - II_14<Age<20 | 3.30 | 0.00 | 0.01 |
| +1/NA (mm) | I_0<Age<13 - II_Age>21 | 3.29 | 0.00 | 0.02 |
| +1/NA (mm) | I_Age>21 - II_0<Age<13 | 3.19 | 0.00 | 0.02 |
| +1/NA (mm) | I_Male_0<Age<13 - II_Female_0<Age<13 | 5.13 | 0.00 | 0.00 |
| +1/NA (mm) | I_Male_0<Age<13 - II_Female_14<Age<20 | 3.71 | 0.00 | 0.01 |
| +1/NA (mm) | I_Male_0<Age<13 - II_Male_0<Age<13 | 3.58 | 0.00 | 0.02 |
| +1/NA (mm) | I_Female_0<Age<13 - II_Female_0<Age<13 | 3.48 | 0.00 | 0.03 |
| -1/ML angle | I_Male - II_Female | 4.92 | 0.00 | 0.00 |
| -1/ML angle | I_Female - II_Male | 3.07 | 0.00 | 0.01 |
| -1/ML angle | I_0<Age<13 - II_14<Age<20 | 4.54 | 0.00 | 0.00 |
| -1/ML angle | I_Male_0<Age<13 - II_Female_0<Age<13 | 3.79 | 0.00 | 0.01 |
| -1/ML angle | I_Male_0<Age<13 - II_Male_0<Age<13 | 3.70 | 0.00 | 0.01 |

**S. Table 5:** Significant differences in dental parameters between different skeletal classes, considering age and gender subgroups, according to Kruskal Wallis with post hoc Dunn analysis and Bonferroni correction. Statistical significance was set at p < 0.01 and p < 0.05. Z = difference between the mean ranks of each two groups, p.unadj = unadjusted p-value in Dunn's test, p adj = adjusted p-value (Bonferroni).

**S. Table 6:** Significant differences in Calculated_ANB between different skeletal classes

| **parameter** | **Group comparison** | **Z-score** | **P. unadj** | **p adj** |
| --- | --- | --- | --- | --- |
| Calculated_ANB | I_Male - II_Female | -14.36 | 0.00 | 0.00 |
| Calculated_ANB | I_Female - II_Male | -13.44 | 0.00 | 0.00 |
| Calculated_ANB | I_14<Age<20 - II_0<Age<13 | -11.70 | 0.00 | 0.00 |
| Calculated_ANB | I_0<Age<13 - II_14<Age<20 | -9.16 | 0.00 | 0.00 |
| Calculated_ANB | I_Age>21 - II_0<Age<13 | -7.05 | 0.00 | 0.00 |
| Calculated_ANB | I_Age>21 - II_14<Age<20 | -6.10 | 0.00 | 0.00 |
| Calculated_ANB | I_0<Age<13 - II_Age>21 | -6.07 | 0.00 | 0.00 |
| Calculated_ANB | I_14<Age<20 - II_Age>21 | -5.91 | 0.00 | 0.00 |
| Calculated_ANB | I_Male_0<Age<13 - II_Female_0<Age<13 | -12.84 | 0.00 | 0.00 |
| Calculated_ANB | I_Female_0<Age<13 - II_Female_0<Age<13 | -12.63 | 0.00 | 0.00 |
| Calculated_ANB | I_Male_0<Age<13 - II_Male_0<Age<13 | -11.82 | 0.00 | 0.00 |
| Calculated_ANB | I_Female_0<Age<13 - II_Male_0<Age<13 | -11.53 | 0.00 | 0.00 |
| Calculated_ANB | I_Female_14<Age<20 - II_Female_0<Age<13 | -9.20 | 0.00 | 0.00 |
| Calculated_ANB | I_Female_14<Age<20 - II_Male_0<Age<13 | -8.93 | 0.00 | 0.00 |
| Calculated_ANB | I_Male_14<Age<20 - II_Female_0<Age<13 | -7.41 | 0.00 | 0.00 |
| Calculated_ANB | I_Male_0<Age<13 - II_Female_14<Age<20 | -7.35 | 0.00 | 0.00 |
| Calculated_ANB | I_Male_14<Age<20 - II_Male_0<Age<13 | -7.27 | 0.00 | 0.00 |
| Calculated_ANB | I_Female_0<Age<13 - II_Female_14<Age<20 | -6.98 | 0.00 | 0.00 |
| Calculated_ANB | I_Female_14<Age<20 - II_Female_14<Age<20 | -6.59 | 0.00 | 0.00 |
| Calculated_ANB | I_Male_0<Age<13 - II_Male_14<Age<20 | -5.86 | 0.00 | 0.00 |
| Calculated_ANB | I_Female_14<Age<20 - II_Male_14<Age<20 | -5.63 | 0.00 | 0.00 |
| Calculated_ANB | I_Female_0<Age<13 - II_Male_14<Age<20 | -5.55 | 0.00 | 0.00 |
| Calculated_ANB | I_Male_14<Age<20 - II_Female_14<Age<20 | -5.52 | 0.00 | 0.00 |
| Calculated_ANB | I_Female_Age>21 - II_Male_0<Age<13 | -5.23 | 0.00 | 0.00 |
| Calculated_ANB | I_Female_Age>21 - II_Female_0<Age<13 | -5.21 | 0.00 | 0.00 |
| Calculated_ANB | I_Male_14<Age<20 - II_Male_14<Age<20 | -4.85 | 0.00 | 0.00 |
| Calculated_ANB | I_Male_Age>21 - II_Male_0<Age<13 | -4.76 | 0.00 | 0.00 |
| Calculated_ANB | I_Male_Age>21 - II_Female_0<Age<13 | -4.73 | 0.00 | 0.00 |
| Calculated_ANB | I_Male_0<Age<13 - II_Male_Age>21 | -4.68 | 0.00 | 0.00 |
| Calculated_ANB | I_Female_14<Age<20 - II_Male_Age>21 | -4.68 | 0.00 | 0.00 |
| Calculated_ANB | I_Female_Age>21 - II_Female_14<Age<20 | -4.52 | 0.00 | 0.00 |
| Calculated_ANB | I_Female_0<Age<13 - II_Male_Age>21 | -4.43 | 0.00 | 0.00 |
| Calculated_ANB | I_Female_Age>21 - II_Male_14<Age<20 | -4.32 | 0.00 | 0.00 |
| Calculated_ANB | I_Female_14<Age<20 - II_Female_Age>21 | -4.22 | 0.00 | 0.00 |
| Calculated_ANB | I_Male_0<Age<13 - II_Female_Age>21 | -4.22 | 0.00 | 0.00 |
| Calculated_ANB | I_Male_Age>21 - II_Female_14<Age<20 | -4.21 | 0.00 | 0.00 |
| Calculated_ANB | I_Male_14<Age<20 - II_Male_Age>21 | -4.11 | 0.00 | 0.00 |
| Calculated_ANB | I_Male_Age>21 - II_Male_14<Age<20 | -4.10 | 0.00 | 0.00 |
| Calculated_ANB | I_Female_0<Age<13 - II_Female_Age>21 | -3.95 | 0.00 | 0.01 |
| Calculated_ANB | I_Male_14<Age<20 - II_Female_Age>21 | -3.62 | 0.00 | 0.02 |
| Calculated_ANB | I_Female_Age>21 - II_Female_Age>21 | -3.48 | 0.00 | 0.03 |
| Calculated_ANB | I_Male_Age>21 - II_Female_Age>21 | -3.37 | 0.00 | 0.05 |

**S. Table 6:** Significant differences in Calculated_ANB between different skeletal classes, considering age and gender subgroups, according to Kruskal Wallis with post hoc Dunn analysis and Bonferroni correction. Statistical significance was set at p < 0.01 and p < 0.05. Z = difference between the mean ranks of each two groups, p.unadj = unadjusted p-value in Dunn's test, p adj = adjusted p-value (Bonferroni).

**S. Table 7*:*** Separately analysis of skeletal class I and II for subgroup-differences

| **parameter** | **Group comparison** | **Z-score** | **P. unadj** | **p adj** |
| --- | --- | --- | --- | --- |
| **Skeletal class I** | | | | |
| Go-Me (mm) | I_0<Age<13 - I_14<Age<20 | -6.40 | 0.00 | 0.00 |
| Go-Me (mm). | I_Female_0<Age<13 - I_Male_14<Age<20 | -6.31 | 0.00 | 0.00 |
| S-N (mm) | I_Female_0<Age<13 - I_Male_14<Age<20 | -4.89 | 0.00 | 0.00 |
| S-N (mm) | I_Female - I_Male | -4.22 | 0.00 | 0.00 |
| Go-Me (mm) | I_Male_0<Age<13 - I_Male_14<Age<20 | -4.61 | 0.00 | 0.00 |
| Go-Me (mm) | I_Female_0<Age<13 - I_Female_14<Age<20 | -4.48 | 0.00 | 0.00 |
| Go-Me (mm) | I_Female - I_Male | -3.11 | 0.00 | 0.00 |
| S-N (mm) | I_Female_0<Age<13 - I_Male_0<Age<13 | -3.63 | 0.00 | 0.00 |
| S-N (mm) | I_0<Age<13 - I_14<Age<20 | -3.19 | 0.00 | 0.00 |
| SNPg angle | I_0<Age<13 - I_14<Age<20 | -2.85 | 0.00 | 0.01 |
| SNB angle | I_0<Age<13 - I_14<Age<20 | -2.51 | 0.01 | 0.04 |
| Wits appraisal | I_Female - I_Male | -2.00 | 0.05 | 0.046 |
| +1/NA (mm) | I_Female - I_Male | -2.15 | 0.03 | 0.03 |
| **Skeletal class II** | | | | |
| Go-Me (mm) | II_0<Age<13 - II_Age>21 | -5.15 | 0.00 | 0.00 |
| S-N (mm) | II_0<Age<13 - II_Age>21 | -4.03 | 0.00 | 0.00 |
| Go-Me (mm) | II_Female_0<Age<13 - II_Female_Age>21 | -4.15 | 0.00 | 0.00 |
| S-N (mm) | II_Female_0<Age<13 - II_Male_Age>21 | -3.68 | 0.00 | 0.00 |
| Go-Me (mm) | II_Female_0<Age<13 - II_Male_Age>21 | -3.67 | 0.00 | 0.00 |
| Wits appraisal | II_Female - II_Male | -2.74 | 0.01 | 0.01 |
| Go-Me (mm) | II_0<Age<13 - II_14<Age<20 | -3.05 | 0.00 | 0.01 |
| Go-Me (mm) | II_Female_Age>21 - II_Male_0<Age<13 | 3.47 | 0.00 | 0.01 |
| S-N (mm) | II_0<Age<13 - II_14<Age<20 | -2.94 | 0.00 | 0.01 |
| Go-Me (mm) | II_14<Age<20 - II_Age>21 | -2.76 | 0.01 | 0.02 |
| Go-Me (mm) | II_Male_0<Age<13 - II_Male_Age>21 | -3.05 | 0.00 | 0.03 |

**S. Table 7*:*** Subgroup differences within skeletal class II and I patients with a statistical difference according to Kruskal Wallis with post hoc Dunn analysis and Bonferroni correction. The post hoc test was done separately for each skeletal class. Statistical significance was set at p < 0.01 and p < 0.05. Z = difference between the mean ranks of each two groups, p.unadj = unadjusted p-value in Dunn's test, p adj = adjusted p-value (Bonferroni).

**S. Figure 1:**


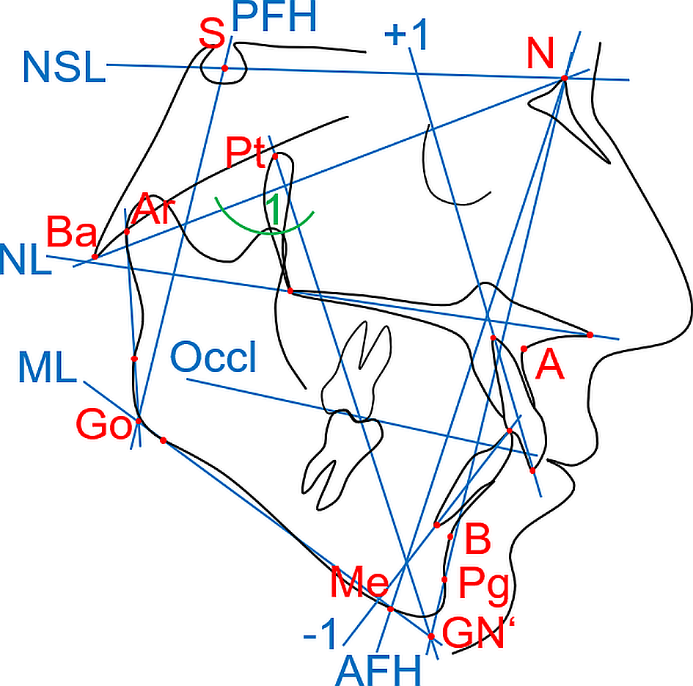


**S. Figure 1:** Reference lines and marks needed for cephalometric evaluation. Angle 1 = Facial axis. Details are described in Table 1.
